# Concordance between cancer gene alterations in tumor and circulating tumor DNA correlates with poor survival in a real‐world precision‐medicine population

**DOI:** 10.1002/1878-0261.13383

**Published:** 2023-03-25

**Authors:** Shai Rosenberg, Gil Ben Cohen, Shumei Kato, Ryosuke Okamura, Scott M. Lippman, Razelle Kurzrock

**Affiliations:** ^1^ Gaffin Center for Neuro‐Oncology, Sharett Institute for Oncology, Hadassah Medical Center and Faculty of Medicine Hebrew University of Jerusalem Israel; ^2^ The Wohl Institute for Translational Medicine, Hadassah Medical Center and Faculty of Medicine Hebrew University of Jerusalem Israel; ^3^ Center for Personalized Cancer Therapy, Moores Cancer Center University of California San Diego La Jolla CA USA; ^4^ Department of Surgery Kyoto University Hospital Japan; ^5^ WIN Consortium and Medical College of Wisconsin Milwaukee WI USA

**Keywords:** cancer, circulating DNA, genomics, survival, tissue DNA

## Abstract

Genomic analysis, performed on tumoral tissue DNA and on circulating tumor DNA (ctDNA) from blood, is the cornerstone of precision cancer medicine. Herein, we characterized the clinical prognostic implications of the concordance of alterations in major cancer genes between tissue‐ and blood‐derived DNA in a pan‐cancer cohort. The molecular profiles of both liquid (Guardant Health) and tissue (Foundation Medicine) biopsies from 433 patients were analyzed. Mutations and amplifications of cancer genes scored by these two tests were assessed. In 184 (42.5%) patients, there was at least one mutual gene alteration. The mean number of mutual gene‐level alterations in the samples was 0.67 per patient (range: 0–5). A higher mutual gene‐level alteration number correlated with shorter overall survival (OS). As confirmed in multivariable analysis, patients with ≥2 mutual gene‐level alterations in blood and tissue had a hazard ratio (HR) of death of 1.49 (95% confidence interval [CI]=1–2.2; *P*=0.047), whereas patients with ≥3 mutual gene‐level alterations had an HR of death 2.38 (95% CI=1.47–3.87; *P*=0.0005). Together, our results show that gene‐level concordance between tissue DNA and ctDNA analysis is prevalent and is an independent factor predicting significantly shorter patient survival.

## Graphical abstract

The molecular profiles of both liquid (circulating tumor DNA [ctDNA] from blood) and tissue (tumor) biopsies from 433 patients with diverse cancers were compared via next‐generation sequencing. Gene‐level concordance between tissue DNA and blood ctDNA analysis was prevalent and higher concordance was an independent factor predicting significantly shorter patient survival.

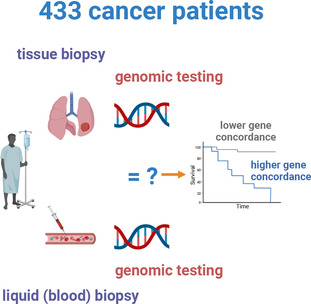

**Abbreviations**
CHIP: clonal hematopoiesis of indetermined potential
ctDNA: circulating tumor DNA
FFPE: formalin‐fixed, paraffin‐embedded
HR: hazard ratio
MB: megabase
NGS: next generation sequencing
OS: overall survival
TMB: tumor mutational burden
VAF: variant allele fraction
VUS: variants of unknown significance

## 1 Introduction

Malignant transformation occurs, at least in part, due to acquired somatic genomic alterations [1], as identified by large‐scale projects depicting the molecular portfolios of many cancers [2, 3, 4]. These projects enabled the realization that most metastatic cancers have complex mutational landscapes that are distinct from each other [5, 6]. Further, over the last few years, rapid advances in sequencing technology made tumor genomic evaluation feasible in the clinical setting. Simultaneously, many novel compounds targeting specific tumor gene products have been approved, leading to the development of a precision medicine strategy for management of malignancies [7, 8, 9]. This strategy has yielded important clinical breakthroughs, such as targeting *ALK* rearrangements in lung cancer [10] and *NTRK* fusions across cancers [11], among others [12].

Conventionally, genomic evaluation of cancers is performed on tissue DNA from tumor specimens obtained by invasive biopsy or surgery. Circulating tumor DNA (ctDNA) reflects cancer‐derived DNA that is shed into the bloodstream. These molecules can be isolated from a small blood sample and assessed for genomic alterations (“liquid biopsy”) [13]. The advantages of liquid biopsy, in addition to the fact that acquiring the blood sample is not invasive, is that it can be obtained in cases in which tumor material is difficult to access or is not sufficient for molecular profiling; moreover, serial samples can be exploited to monitor the response to treatment, and to detect the emergence of mutations that drive resistance and/or that may need to be targeted by other drugs [14, 15, 16, 17].

The concordance level between genomic analysis of tumor tissue DNA and blood‐derived ctDNA when acquired at the same time can be as high as 80–90% [18, 19, 20, 21], although other reports have found lower levels of concordance [15]. A high positive overall blood and tissue concordance rate may be driven in some cases by the large negative/negative subset [22]. It should also be noted that there may be biologic reasons that underlie differences between tissue DNA and blood‐derived ctDNA results: (a) tissue DNA sequencing evaluates the genomics in a small sample of tissue, while ctDNA may uncover alterations from DNA released into the bloodstream from multiple metastatic sites, better mirroring cancer heterogeneity [15]: (b) ctDNA can be suppressed by treatment; and (c) shedding of DNA from cancer cells into the circulation may be limited from some metastatic or primary cancer sites, especially if the tumor burden is low.

Herein, we examined the concordance between blood‐derived ctDNA alterations and tissue DNA aberrations, as determined by next‐generation sequencing (NGS). We demonstrate that higher concordance between tissue and blood DNA alterations is independently associated with poorer outcomes.

## 2 Materials and methods

### 2.1 Patients

We included 433 patients seen at the University of California San Diego (UCSD) Moores Cancer Center. The patients had a variety of advanced cancers and both prior systemic therapies or no prior therapies. Each patient had both tissue and ctDNA assessed. The samples collection started in June 2014 and the last ctDNA specimen was obtained on 09/2017. Eligibility for inclusion implied adequate follow up, and demographic information availability. Demographics of each of these patients was curated from the electronic medical record. The study methodologies conformed to the standards set by the Declaration of Helsinki and experimental interventions were approved by the UCSD Internal Review Board‐approved protocol (NCT02478931) and included any investigational procedures for which the participant provided written informed consent.

### 2.2 Next‐Generation sequencing (NGS)

Tissue NGS testing was analyzed by Foundation Medicine (https://www.foundationmedicine.com/genomic‐testing#how‐does‐it‐work); blood‐derived ctDNA molecular NGS was evaluated by Guardant Health(http://www.guardanthealth.com/). The Foundation Medicine test is now FDA‐approved for reporting deleterious variants. Both laboratories are Clinical Laboratory Improvement Amendment (CLIA)‐accredited. Detailed methods were previously reported [23, 24].

#### 2.2.1 Variants of unknown significance

Synonymous alterations and other variants of unknown significance (VUS) were excluded and only characterized deleterious alterations were included in this report [23]. Characterized alterations refer to pathogenic alterations that are not VUSs.

#### 2.2.2 Tissue NGS

Tissue NGS was assessed at Foundation Medicine (clinical‐grade CLIA testing) with assay panels of 236 or 315 genes. Detailed methods are as previously reported (Cambridge, MA, www.foundationmedicine.com) [23]. All tissue samples (formalin‐fixed, paraffin‐embedded [FFPE]) were reviewed by a pathologist to ensure that ≥20% of the nuclei in the sample were derived from the tumor, a sample volume of ≥1 mm^3^, and nucleated cellularity ≥80% or ≥30,000 cells. Mean sequencing depth was >250×, with 100× at >99% of exons. This method of sequencing permits identification of copy number alterations, somatic mutations, and gene rearrangements, with >99% sensitivity for base substitutions at ≥5% mutant allele frequency and 99% specificity, as well as >95% sensitivity for copy number alterations. A threshold of ≥8 copies for gene amplification was utilized.

For tumor mutational burden (TMB), measured in mutations per megabase, the number of somatic mutations detected by NGS (interrogating 1.2 mb of the genome) were quantified and that value extrapolated to the whole exome utilizing a validated algorithm [25]. Oncogenic driver alterations and germline polymorphisms were excluded. TMB was measured in mutations per megabase (mb).

#### 2.2.3 Blood‐derived ctDNA NGS

As published by Lanman *et al*., 5–30 ng of ctDNA was isolated from plasma and sequencing libraries were made with custom in‐line barcode molecular tagging and complete sequencing at 15,000× read depth [24]. The panels utilize hybrid capture followed by NGS of the crucial exons in a panel of 54–73 genes, and read out all four major types of genomic alterations: fusions, point mutations, indels, and copy number amplifications. Postsequencing bioinformatics matches the complementary strands of each barcoded DNA fragment to remove false‐positive results [24]. The variant allele fraction (VAF, which equals %ctDNA) is calculated as the number of mutated DNA molecules divided by the total number (wildtype plus mutate) of DNA fragments at that allele. We analyzed the maximum %ctDNA of any deleterious variant. Most cell‐free DNA is wildtype; therefore, the median %ctDNA of somatic alterations is <0.5%. The analytic sensitivity reaches detection of one to two single‐mutant fragments from a 10‐mL blood sample (0.1% is the threshold for detection), and the analytic specificity is >99.9999% [24].

#### 2.2.4 Genes tested

Guardant 360 panel tested before February 2015 included 54 genes. All samples analyzed before February 2015 were analyzed by this 54‐gene panel. Samples tested afterwards were analyzed by different panel versions that included 68–73 genes in ctDNA analysis. Sixty‐three genes were mutual to all of these later panels and others were excluded from the analysis. We analyzed genes that were sequenced in both the tissue biopsy and the liquid biopsy and excluded all other genes. The Foundation One tissue panel included 309 genes. Intersection with the 54 genes in the first panel resulted in 53 genes. For three of these genes, amplifications were measured and included. We examined the intersection of Foundation Medicine tissue genes with the 63 ctDNA genes tested in the panels after February 2015. Altogether, 55 genes were available for which both ctDNA and tissue DNA was analyzed on February 2015 and later. Sixteen of these genes were also tested for amplifications, which we included. Table S1 provides the two gene lists.

#### 2.2.5 Definition of genomic alterations concordance type

Genes that were not analyzed by both ctDNA and by tissue were not included in the analysis. For each participant, the specific genomic anomaly (or their absence) was compared between tissue and ctDNA. For each genetic abnormality in tissue DNA, we examined whether an abnormality in the same gene was discerned in ctDNA and also whether this genetic abnormality was identical (at the same gene locus) to that found in the tissue DNA.

We defined gene‐level concordance as positive concordance when the gene alteration is found in both tests (not necessarily the same locus aberration) and negative concordance otherwise. We defined mutation‐level concordance as positive concordance when exactly the same locus aberration was identified in both tests (e.g. same location in a single nucleotide variant [point mutation] or amplification in both, etc.) and negative concordance otherwise.

### 2.3 Statistical analysis

Statistical analysis was done with R programming language version 3.5.1 [26]. Survival time was calculated from time of diagnosis and the defined event was death. Survival was analyzed by Cox regression; patients still alive at the last follow up were censored at that time. Cox proportional hazards regression model and multivariable analysis were performed using the survival r package [27]. Visualization was performed by the r packages of ggplot2 [28]; forest plots (blobbograms) were calculated and drawn by the r package of forest model [29]. Balloon plots (representing the residuals of Chi‐square test) were done with the r package of corrplot [30]. The graphical abstract was created by BioRender.com.

## 3 Results

### 3.1 Cohort characteristics

Our cohort included 433 individuals with several different tumor types as described in Rosenberg *et al*. [31]. All but 28 patients had advanced disease that was surgically unresectable and/or metastatic. For each patient, genomic analysis was carried out twice: (a) for DNA obtained from tissue biopsy (Foundation One test) and (b) for ctDNA (Guardant 360 test). The median time between the tissue biopsy date and the blood draw for ctDNA was 3.7 months (range: −14.6–241.4). The median age at the time of ctDNA liquid biopsy was 62 years (range: 19–93 years) and the median age at diagnosis was 58.6 years (range: 2.2–90.5 years). Approximately 55% of patients were women. The most common tumor types included gastrointestinal cancers, lung cancers, breast and gynecologic cancers, and head and neck cancers [31]. The most common gene mutated in both blood and tissue biopsies was *TP53*.

### 3.2 Genomic landscape

There were 853 genomic alterations in the tissue DNA analysis and of these, 739 were point mutations and 114 were amplifications. The mean number of alterations per patient was 1.97 (range: 0–8). There were 680 genomic alterations in the ctDNA analysis and of these, 466 were point mutations and 214 were amplifications. The mean number of alterations per patient was 1.57 (range: 0–15). Table 1 provides the frequency of gene alterations in the cohort.

**Table 1:** Number of mutated gene alterations in tissue DNA and ctDNA along with gene and mutation level alteration concordance. For example, for the *TP53* gene there is a total of 196 tumors with tissue DNA alterations and 158 tumors with ctDNA alterations. There are 110 tumors for which a *TP53* alteration was found in both tissue DNA and ctDNA, but not necessarily in the same positions. There are 69 tumors for which a *TP53* alteration in the same locus was identified in both tissue DNA and ctDNA.

Gene	Tissue DNA alteration number	ctDNA alteration number	Mutual gene alteration number	Mutual mutation alteration number
TP53	196	158	110	69
KRAS	81	56	36	11
EGFR	45	43	16	6
PIK3CA	44	42	19	6
APC	40	20	16	14
MYC	28	25	8	3
PTEN	26	12	10	9
ERBB2	23	15	8	6
CDKN2A	22	8	6	5
ARID1A	20	7	3	3
SMAD4	18	9	6	4
BRAF	17	31	8	3
IDH1	17	2	1	0
FBXW7	11	3	2	2
BRCA2	10	3	1	1
GNAS	10	9	3	2
NF1	10	9	1	1
CTNNB1	9	6	4	3
ATM	8	7	1	1
CCNE1	8	16	3	1
ESR1	8	5	4	2
FGFR1	8	12	4	2
MET	8	23	4	2
CDK4	7	3	1	1
KIT	6	6	1	2
FGFR2	5	2	2	1
NRAS	5	4	1	1
AKT1	4	2	1	1
FGFR3	4	1	1	1
MAP2K1	4	2	2	2
VHL	4	3	2	1
HRAS	2	2	1	1
GNA11	1	1	1	1
GNAQ	1	1	1	1

### 3.3 Gene‐level and mutation‐level alteration concordance in blood and tissue

We measured the gene‐level concordance in the cohort. In 249 (57.5%) patients, there were no mutual gene alterations in tissue DNA and ctDNA analysis. In the remaining 184 patients, there was at least one mutual gene alteration. The mean number of mutual gene‐level alterations in the samples was 0.67 per patient (range: 0–5). The number of concordant samples for each concordant gene alteration is given in Table 1. There was a significant correlation between the number of mutual gene‐level alterations and the number of tissue DNA genes with a pathogenic mutation (0.46, *P* < 2.2e‐16, Pearson correlation test), but not with tissue tumor mutation burden (TMB) (0.044, *P*=0.37, Pearson correlation test). There was a negative correlation between the time between tissue DNA and ctDNA testing and the number of mutual genes (−0.18, *P*=0.0001363, Pearson correlation test, Fig. S1). There was a positive correlation between percent ctDNA and tissue gene number (*R*=0.17, *P*=0.0004) but not with TMB (*R*=0.08, *P*=0.09). Stronger correlation was identified between percent ctDNA and mutual gene number (*R*=0.44, *P* < 2.2e‐16), Fig. S2. The number of mutual gene‐level alterations differed markedly between tumor types (*P*=0.0004, Chi‐square test). Gastrointestinal, breast and gynecologic cancer were associated with a larger number of mutual gene‐level alterations in tissue and blood, whereas brain tumors were associated with a low number of mutual gene‐level alterations in tissue and blood (Fig. S3a).

We also measured the mutation‐level alteration (gene locus) specific concordance in the cohort. The mean number of exact mutual mutation‐level alterations was 0.6 per patient (range: 0–5). There was a significant correlation between the number of mutual mutation‐level alteration number and the number of tissue DNA genes with pathogenic mutations (0.46, *P* < 2.2e‐16, Pearson correlation test), but not with tissue TMB (0.03, *P*=0.54, Pearson correlation test). The number of mutual mutation‐level alterations differed markedly between tumor types (*P*=7.4e‐06, Chi‐square test). Similar to the gene‐level alteration concordance above gastrointestinal, breast, and gynecologic cancer were associated with a larger number of mutual mutation‐level alteration concordance between blood and tissue, whereas brain tumors were associated with a low number of mutual mutation‐level alterations, Fig. S3b.

**Fig. 1:**
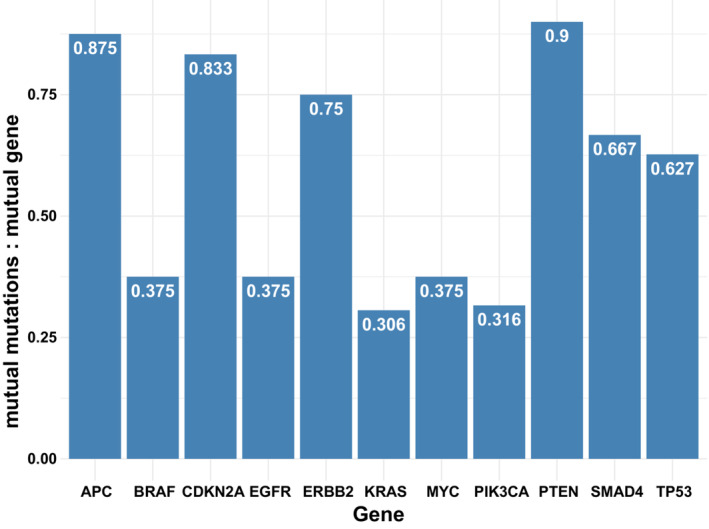
The frequency of mutation‐level concordance given gene‐level concordance. Only genes with gene concordance in more than five samples are shown. Gene‐level concordance implies that the same gene (but not necessarily the same locus) is aberrant in both ctDNA and tissue; mutation‐level concordance implies that the same locus in the same gene is aberrant in both ctDNA and tissue.

We estimated the chance of mutual mutation‐level alteration concordance given mutual gene‐level concordance for genes with five or more concordant samples (11 genes, Fig. 1). The median frequency was 0.63 (range: 0.306–0.875).

### 3.4 Gene‐level and mutation‐level (locus level) alteration concordance in blood and tissue were predictive of shorter survival in multivariate analysis

**Fig. 2:**
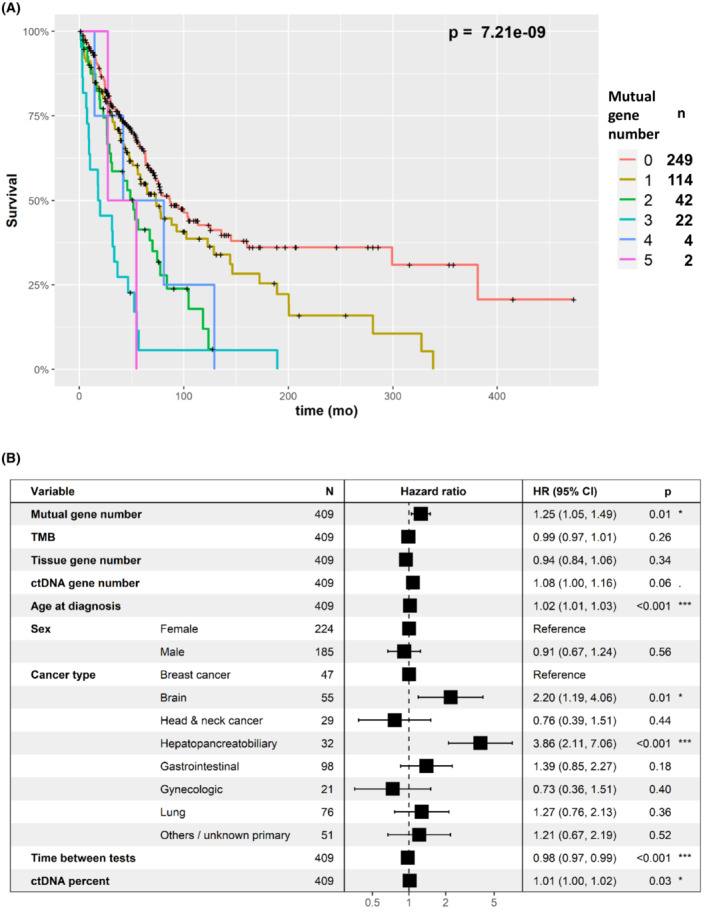
Survival analysis in relation to the number of concordant genes (gene‐level concordance). (A) Kaplan–Meier, univariate analysis. The plots show that the greater the number of gene‐level alteration concordance in tissue and blood ctDNA, the shorter the survival. Number of samples in each mutual gene number category are provided in the right legend. (B) Multivariable analysis (Cox regression). The figure shows that greater gene‐level alteration concordance in tissue and blood (denoted as “mutual gene number”) was associated with significantly shorter survival, independent of other factors. All parameters aside from sex and cancer type were assessed as continuous variables. Younger age, longer time between tests, and lower ctDNA percent was associated with longer survival times, whereas brain and hepatopancreatobiliary cancers were associated with shorter survival times. (Note that the box overlaps the “1” line at times, because of a graphical limitation, although the *P* value [and 95% CI] are significant.) CI, confidence interval; ctDNA, circulating tumor DNA; HR, hazard ratio; TMB, tumor mutational burden. *P* values: *: 0.01–0.05, ***: 0–0.001.

A higher mutual gene‐level alteration number was associated with shorter overall survival (OS) (Fig. 2A, *P*=7.21e‐9, Cox univariate survival test). As shown in Fig. 2B, this was also confirmed in multivariable analysis (hazard ratio [HR]=1.25, *P*=0.014). Other factors associated with shorter OS in the multivariable test were higher age at diagnosis, certain tumor types (brain and hepatopancreatobiliary), shorter time between the tests, and higher percent ctDNA. Importantly, the tumor gene number and ctDNA gene number were also associated with overall survival (*P*=0.021, *P*=1.08E‐5, respectively). A higher percent of ctDNA was also significantly associated with shorter OS (*P*=7.43E‐6) (Fig. S4). However, in the multivariable analysis tumor gene number was not significant and ctDNA gene number was marginally significant (HR=1.08, *P*=0.06). Percent ctDNA was independently significant in the multivariable analysis (HR=1.01, *P*=0.03). These differences between the association of survival with tissue DNA and ctDNA parameters possibly reflect the higher importance of number of ctDNA mutations for survival predictions. Still, the highest level of independent significance was for concordance between tumor and ctDNA alterations and poorer survival (HR=1.25, *P*=0.014, multivariable Cox regression analysis).

Since the time gap between tissue DNA and ctDNA tests is negatively correlated with the mutual gene number, in addition to the multivariable analysis we also performed analysis excluding patients for which the temporal gap was more than 6, 12, 24, and 48 years. Indeed, despite the power reduction due to excluding samples―the *P* values of number of mutual genes and survival was higher. The *P* value for all the data was 0.014. For the filtered datasets: <48 months – 0.008, <24 months – 0.003, <12 months – 0.003, <6 months – 0.002 (Fig. S5). A higher mutual mutation alteration number was also associated with shorter OS (*P*=4.52e‐9, Cox univariate survival test). This was also confirmed in a multivariable analysis (HR=1.25, *P*=0.013, Fig. S6).

**Fig. 3:**
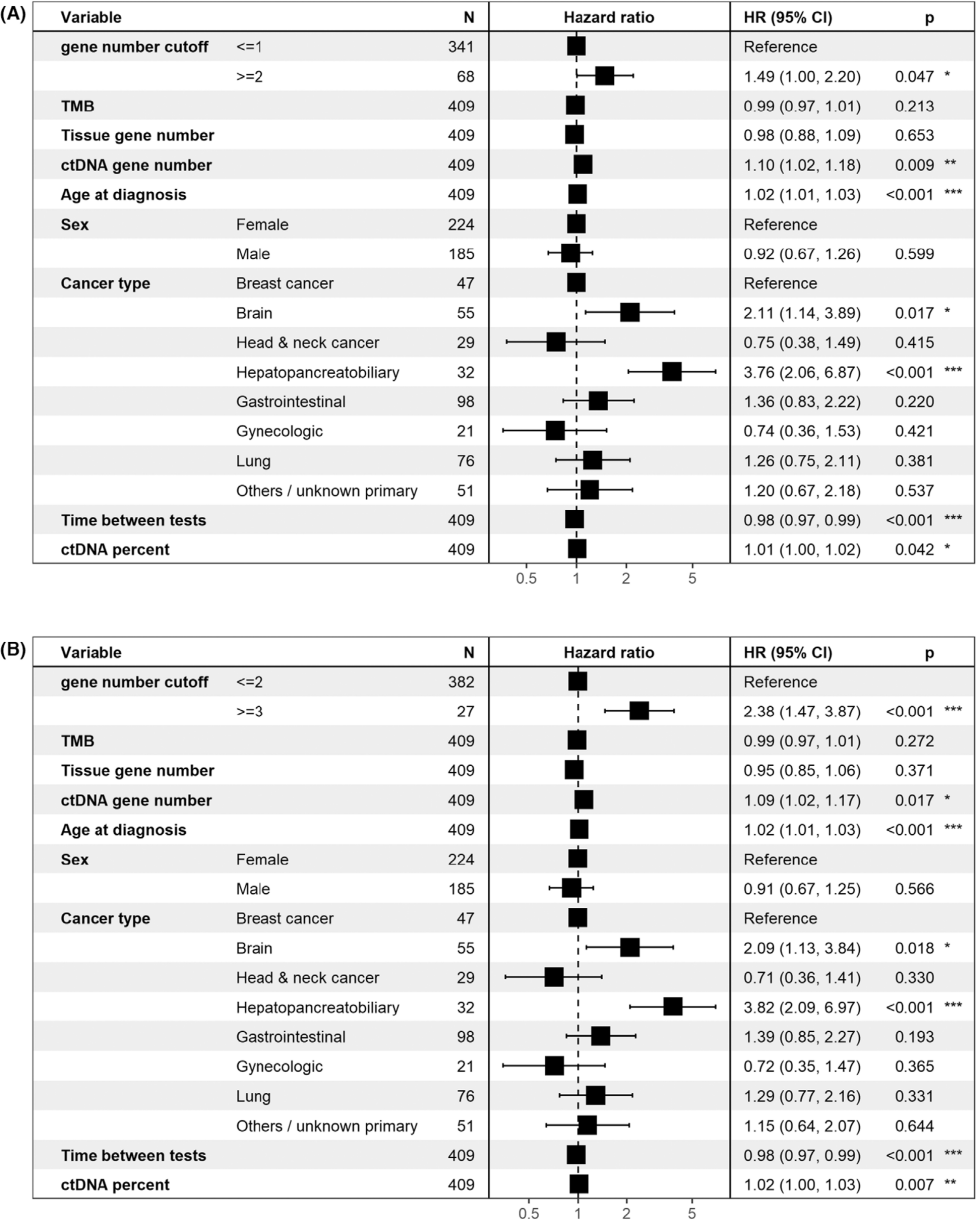
Multivariable survival analysis (Cox regression) with different cutoffs for gene‐level concordance. Regardless, if the cutoff was ≥2 vs <2 or ≥3 vs <3, greater gene‐level concordance is independently associated with shorter survival. (A) Patients with two or more concordant altered genes vs one or zero concordant genes. (B) Patients with three or more concordant genes vs zero to two concordant genes. CI, confidence interval; ctDNA, circulating tumor DNA; HR, hazard ratio; TMB, tumor mutational burden. *P* values: *: 0.01–0.05, **: 0.001–0.01, ***: 0–0.001.

Patients with no mutual gene alteration in blood and tissue had longer OS compared to patients with at least one mutual gene alteration (*P*=6.18e‐6, Cox univariate test), but this was not significant in multivariable analysis (HR=1.14, *P*=0.48). Higher thresholds resulted in significance in the multivariable test: patients with two or more mutual genes had shorter OS compared to patients with zero or one mutual genes (HR=1.49, *P*=0.047). The analysis was even more significant when comparing patients with three or more mutual gene alterations to patients with two or less mutual gene alterations (HR=2.38, *P*=0.0005) see Fig. 3.

**Fig. 4:**
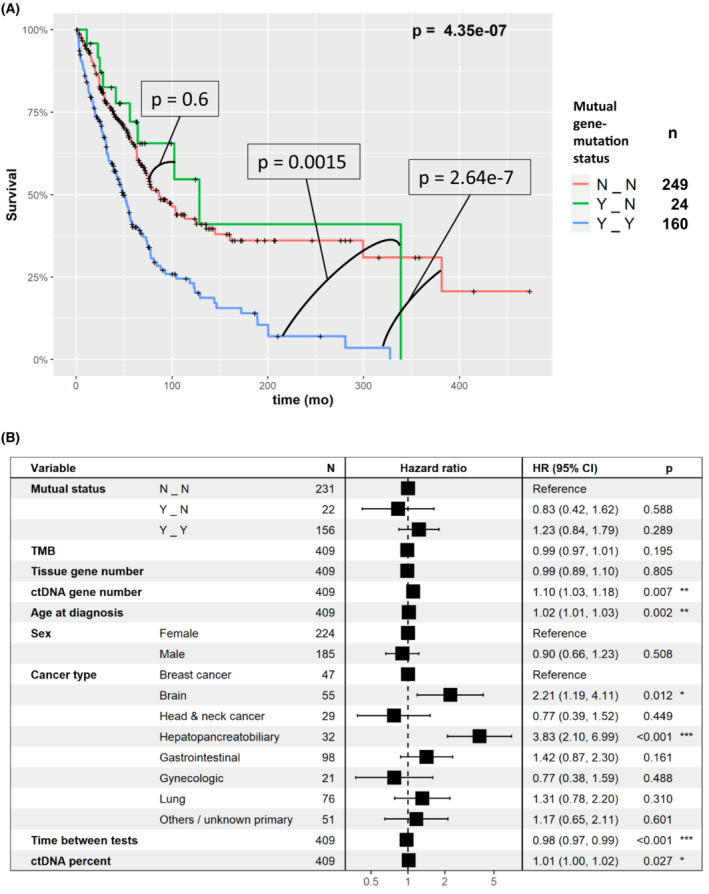
Survival analysis of mutual gene‐level and mutual mutation‐level alteration concordance status between blood and tissue. N_N, no mutual gene‐level nor mutual mutation‐level alteration concordance; Y_N, at least one mutual gene‐level but no mutual mutation‐level alteration concordance; Y_Y, at least one mutual gene‐level and at least one mutual mutation‐level alteration concordance between blood and tissue. (A) Kaplan–Meier, univariate analysis. The plot shows that there is no difference in survival between patients with no mutual gene‐level nor mutual mutation‐level alteration concordance (N_N) and at least one mutual gene‐level but no mutual mutation‐level alteration concordance (Y_N) (*P*=0.6); however, patients with at least one mutual gene‐level and at least one mutual mutation‐level alteration concordance between blood and tissue (Y_Y) have significantly shorter survival than the first two groups (Y_Y vs N_N, *P*=2.64e‐7; Y_Y vs Y_N, *P*=0.0015, Cox univariate test). Examining all three curves confirms this observation (*P* = 4.35e‐7), as shown in the graphic. These results suggest that it is mutation‐level alteration concordance that is important for predicting outcome in univariate analysis. The number of samples in each mutual gene‐mutation status category are provided in the right legend. (B) Multivariable analysis (Cox regression). In multivariable analysis, the difference between the concordance groups was not significant (the lack of impact of mutation level concordance on outcome in multivariable analysis could be attributed to the fact that only 22 tumors had mutual genes but not mutual gene mutation locus concordance). CI, confidence interval; ctDNA, circulating tumor DNA; HR, hazard ratio; TMB, tumor mutational burden. *P* values: *: 0.01–0.05, **: 0.001–0.01, ***: 0–0.001.

**Fig. 5:**
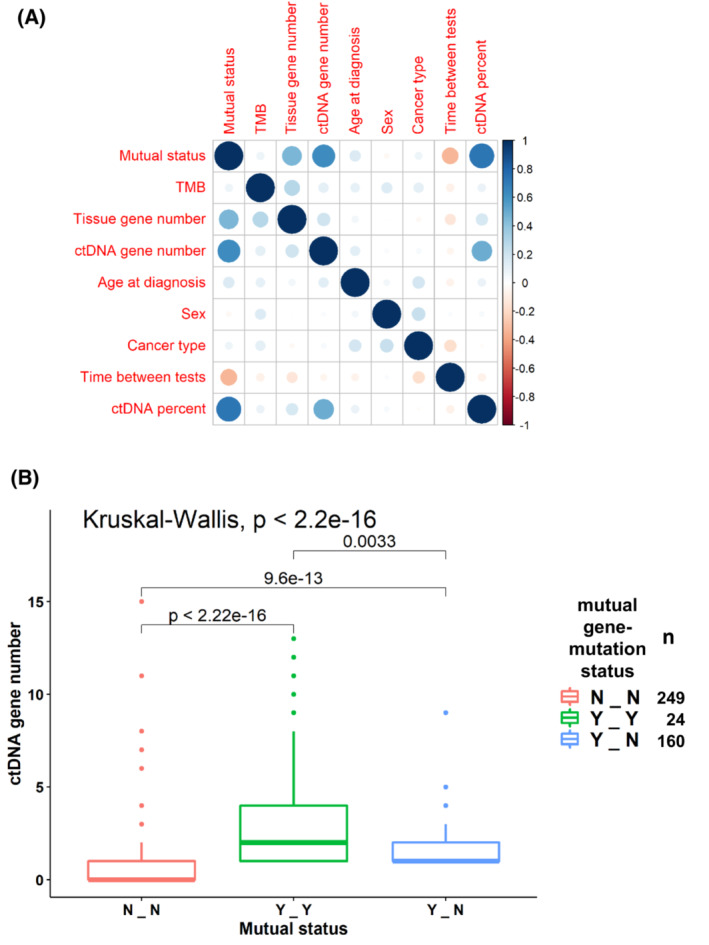
(A) Correlation plot of the independent variables tested. Balloon plot that represents the residuals of the Chi‐square test. Blue implies positive correlation between factors and red implies negative correlation. Intensity of the color reflects the magnitude of the correlation (greater intensity = greater degree of correlation). Mutual status is defined in three levels: N_N, – no mutual gene‐level nor mutual mutation‐level alteration concordance; Y_N, at least one mutual gene‐level but no mutual mutation‐level alteration concordance; Y_Y, at least one mutual gene‐level and at least one mutual mutation‐level alteration concordance between blood and tissue. (B) ctDNA gene number compared to the mutual status of gene‐level and mutation‐level alteration concordance between blood and tissue. Central line represents median value and the box represents the range of 1^st^ to 3^rd^ quartiles. The graphic shows that a higher ctDNA gene alteration number is associated with a greater number of mutual gene‐level alteration concordance (Y‐N) between blood and tissue and an even greater number of mutation‐level alteration concordance between tissue and blood (Y‐Y). Number of samples in each mutual gene‐mutation status category are provided in the right legend. ctDNA, circulating tumor DNA; TMB, tumor mutational burden.

Next, we wanted to examine whether there is a survival difference between patients with gene concordance (and no mutation specific concordance) compared to patients with at least one mutation specific concordance. Importantly, for most samples with at least one mutual gene there was at least one mutual mutation. Hence, there is a paucity of samples (*N*=22) with mutual genes and no mutual mutations compared to samples with both (*N*=156). As shown in Fig. 4, the survival curve of patients with mutual genes and no mutual mutations was similar to the curve of patients without any mutual genes and was significantly longer compared to patients with at least one mutual mutation (*P*=4.35e‐7, Cox univariate test, comparing the three groups). However, this was not significant in the multivariable analysis (HR=1.23, *P*=0.289). In order to identify the major factors that reduced the significance of the test, we repeated testing with exclusion of one factor at a time. Omitting ctDNA gene number was the only exclusion resulting in significance of mutual mutation status (HR=1.48, *P*=0.03). Omitting the time between tests factor resulted in marginal significance of mutual mutations status (HR=1.43, *P*=0.059). Indeed, the ctDNA gene number was highly correlated with mutual mutations status (Fig. 5, *P* < 2.2e‐16, Kruskal–Wallis test) and associated with survival (*P*=1.08e‐5, Cox univariate test).

## 4 Discussion

In this study, we investigated a real‐world cohort of 433 cancer patients for whom two panel genomic tests were performed: tissue DNA and ctDNA. We analyzed only genes and alterations that were sequenced in both tests. We focused on the genes that were mutated in both tests for individual patients' tumors and on genes for which the same locus‐specific alteration was identified in both tests. We aimed to characterize the parameters that were associated with the number of mutual genes and mutual mutations, as well as to investigate the survival implication of these numbers.

We identified that the number of mutual genes per patient was associated with several parameters: several tumor types (gastrointestinal, breast, and gynecologic cancer) showed increased mutual gene alteration numbers in blood and tissue and, as expected, brain tumors were associated with lower numbers of mutual genes, possibly related to less ctDNA shedding in brain tumor patients [14, 32]. Our prior studies have also shown higher concordance rates for blood and tissue gene alterations in specific gastrointestinal malignancies such as colorectal cancer when examining *KRAS* or *TP53* mutations [22, 33].

Importantly, the number of mutual genes altered in blood and tissue was associated with the number of pathogenic mutations in tissue DNA, but not with the general number of alterations as measured by TMB (which in our system does not include deleterious alterations in the TMB count). This finding seems to emphasize that the association of mutual gene alterations in blood and tissue is not related to a higher mutation rate, but rather to the biologic function of the pathogenic variants, perhaps resulting in a selective shedding into the blood for them.

Next, we analyzed the survival implications of gene concordance and identified that the number of concordant gene alterations in blood and tissue correlated with shorter survival independently from other parameters measured. The mere presence of concordant tissue and liquid alterations in one gene was not significantly associated with survival in the multivariable analysis. However, a threshold of two or more mutual gene alterations in tissue and blood was highly significant in predicting a worse survival outcome.

Interestingly, for most of the samples with mutual blood and tissue gene alterations, there were also mutual mutations (meaning that the same locus was altered in the tissue and blood DNA). The correlations between mutual mutation concordance versus mutual gene concordance might be difficult to assess because of the small number of samples in our current study with blood and tissue concordant gene alterations, but discordant locus (mutation) concordance. However, in our prior report examining just *TP53* alterations [31], the presence of *TP53* mutational (locus) concordance in blood and tissue was associated with poor survival independently of other parameters tested. It should be noted that for some genes, such as *TP53*, locus discordance in blood and tissue might be attributable to clonal hematopoiesis of indetermined potential (CHIP) [34].

This study had several limitations. The cohort heterogeneity of tumor types with different mutational landscapes and prognosis may create bias. However, for this reason we carried out multivariable analysis to control for tumor types. In addition, we did not consider the effect of therapies provided to the patients. It is known that different therapies could affect the mutations detected in ctDNA, since some resistant clones are emerging after therapy. However, it should be kept in mind that the therapies received in this group were heterogenous, and this further suggests that the concordance between blood and tissue alterations is a prognostic factor, across tumor types and their management. A third limitation is the variable temporal gap between tissue DNA and ctDNA testing. This could affect the concordance rate, and thus the results as well. This could explain that in 249 (57.5%) patients, no mutual gene alterations in tissue DNA and ctDNA analysis were found. However, it should be pointed out that despite these limitations, and despite the temporal gaps in this real‐world dataset, concordance between tissue DNA and ctDNA still predicted OS. This was independent of time between the tests in a multivariable analysis. A fourth weakness is that we were limited to investigate genes that were sequenced in both tissue DNA and ctDNA testing, and we could only assess point mutations and amplifications. Investigating exome sequencing data might provide a more comprehensive view of the clinical and biological importance of performing both tests. A final limitation is that the technology does not permit analysis of subclones which would require isolation of single cells rather than bulk ctDNA. Importantly, this is a real‐world cohort. While such data provide interesting clinical insights, controlled trials are needed to better characterize the clinical impact of these findings.

## 5 Conclusion

To summarize, in this study we showed that high numbers of genes concordantly mutated in tissue and blood correlate with shorter survival independent of other factors. Larger, prospective clinical trials should be performed in order to establish the clinical utility of the findings presented here.

## Author contributions

SR: Conceptualization, data curation, formal analysis, validation, investigation, visualization, methodology, writing‐original draft. GBC: formal analysis, validation, investigation, writing‐review and editing. SK: Data curation, validation, writing‐review and editing. RO: Data curation, validation, writing‐review and editing. SML: Conceptualization, resources, supervision, funding acquisition, writing‐review and editing. RK: Conceptualization, resources, supervision, funding acquisition, validation, investigation, methodology, writing‐original draft, writing‐review and editing.

## Conflict of interest

Razelle Kurzrock receives research funding from Genentech, Merck Serono, Pfizer, Boehringer Ingelheim, TopAlliance, Takeda, Incyte, Debiopharm, Medimmune, Sequenom, Foundation Medicine, Konica Minolta, Grifols, Omniseq, and Guardant, as well as consultant and/or speaker fees and/or advisory board for X‐Biotech, Neomed, Pfizer, Actuate Therapeutics, Roche, Turning Point Therapeutics, TD2/Volastra, Bicara Therapeutics, Inc.; has an equity interest in IDbyDNA and CureMatch Inc.; serves on the Board of CureMatch and CureMetrix, and is a co‐founder of CureMatch. Scott M. Lippman serves on the Board of Biological Dynamics, and is a co‐founder of io9. Shumei Kato serves as a consultant for Medpace, Foundation Medicine, NeoGenomics and CureMatch. He receives speaker's fee from Roche and Bayer, and advisory board for Pfizer. He has research funding from ACT Genomics, Sysmex, Konica Minolta, OmniSeq and Personalis. Shai Rosenberg is a medical and scientific consultant of Barcode and Oncotest companies, he receives research funding from MSD.

### Peer review

The peer review history for this article is available at https://publons.com/publon/10.1002/1878‐0261.13383.

## Supporting information


**Fig. S1.** Correlation between the time gap between tissue DNA and ctDNA tests and the number of concordant genes.

**Fig. S2.** Correlation plot of tissue DNA and ctDNA parameters.

**Fig. S3.** Balloon plots representing the residuals of the Chi‐square tests of: a. mutual gene concordance number tumor type; b. mutual mutation concordance number tumor type.

**Fig. S4.** Univariate survival analysis of (a) quartiles of percent ctDNA, (b) quartiles of tissue DNA gene number, (c) quartiles of ctDNA gene number.

**Fig. S5.** Multivariable analysis for the survival test in relation to the number of concordant genes (gene‐level concordance), similar to Fig. 2B including only samples for which the time gap between tissue DNA and ctDNA (a) ≤ 48 months, (b) ≤ 24 months, (c) ≤ 12 months, (d) ≤ 6 months.

**Fig. S6.** Survival analysis in relation to the number of concordant mutations (mutation‐level concordance).

**Table S1.** A list of genes compared in the two cohorts of the study.

**Table S2.** (a) Clinical information and sample‐by‐sample comparison of the two platforms, both at a gene level and at a mutation/variation level. (b) Sample information including all genetic alterations (not only the non‐overlapping genes) identified by the tissue biopsy and the ctDNA exams.

## Data Availability

The datasets used and/or analyzed during the current study are provided in Table S2.
