# Supplementary figures and images for: Concordance between cancer gene alterations in tumor and circulating tumor DNA correlates with poor survival in a real‐world precision‐medicine population
Source: Mol Oncol. 2023 Mar 25;17(9):1844–56. doi: 10.1002/1878-0261.13383 (PMC10483598; doi:10.1002/1878-0261.13383)

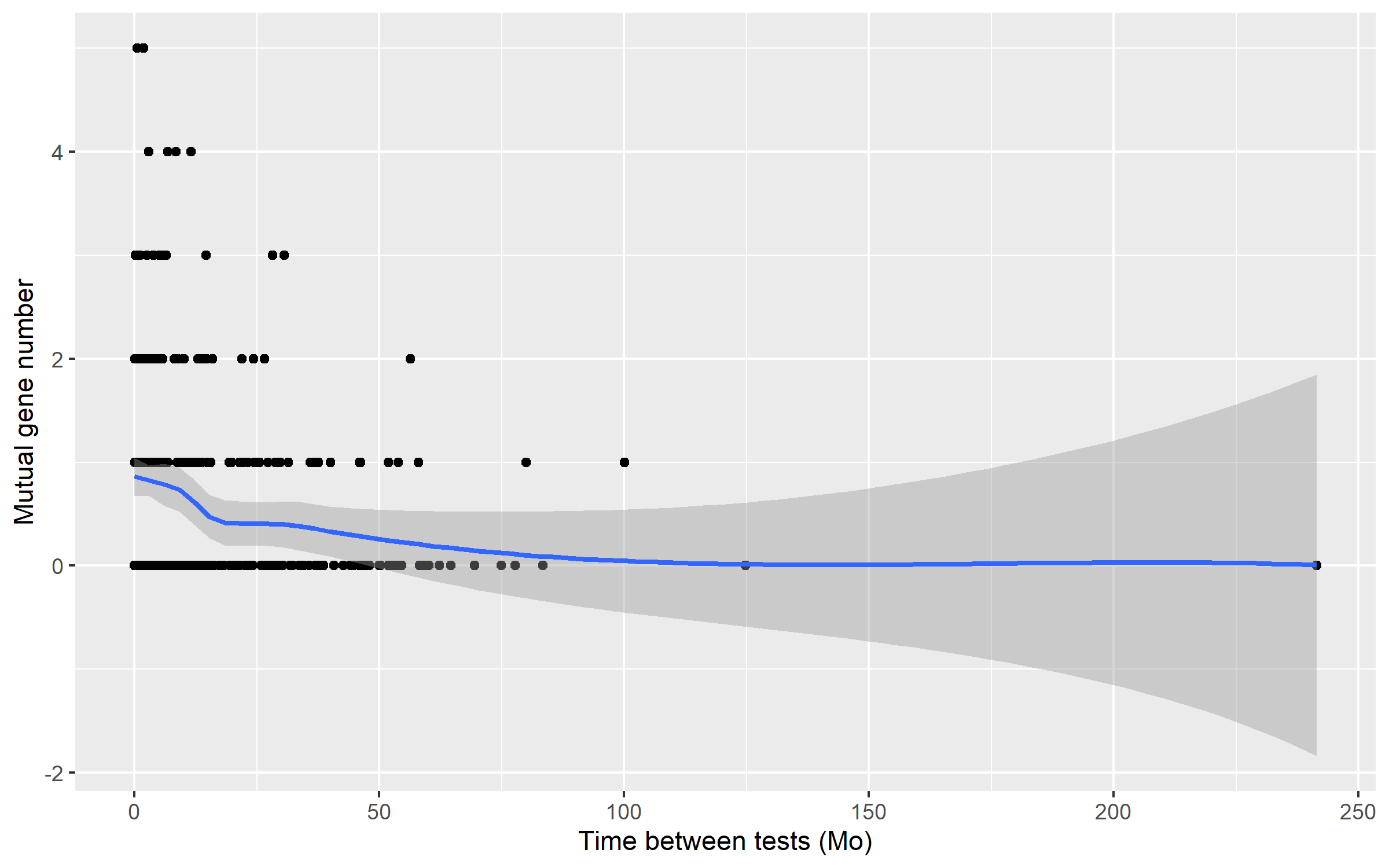

Supplement: Supplementary file 1 — Fig. S1. Correlation between the time gap between tissue DNA and ctDNA tests and the number of concordant genes. [file MOL2-17-1844-s005.tiff]

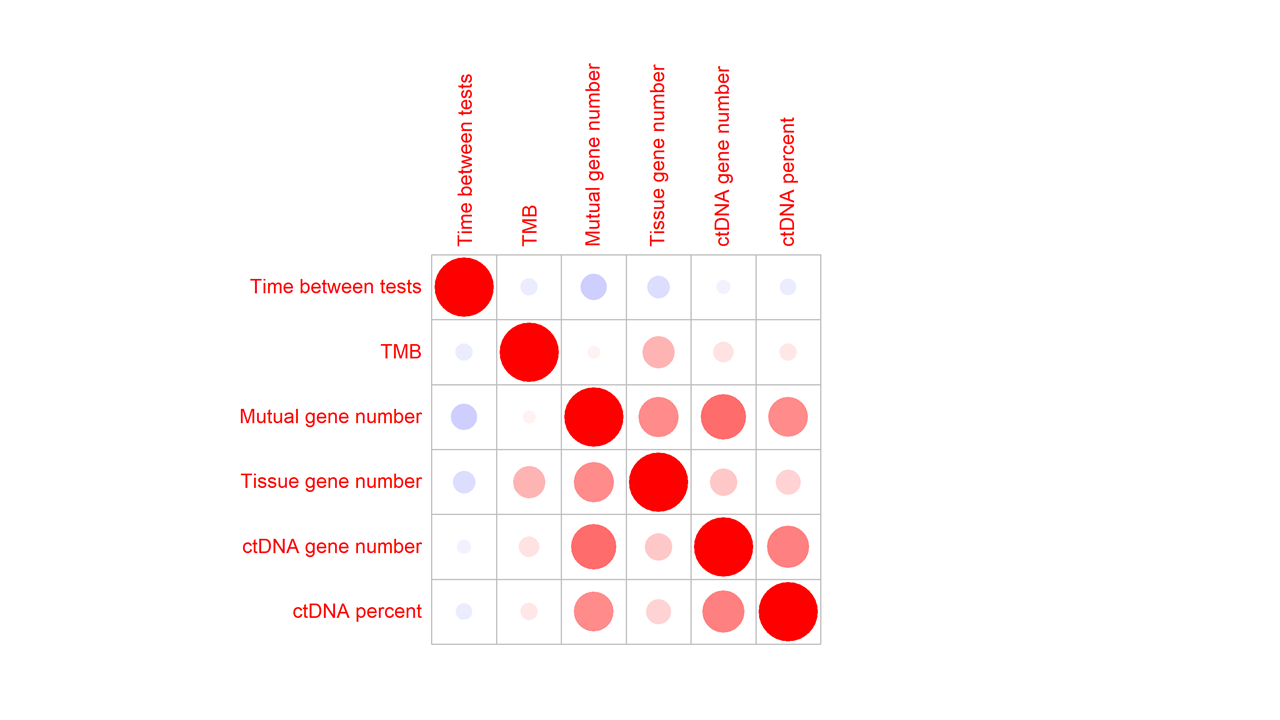

Supplement: Supplementary file 2 — Fig. S2. Correlation plot of tissue DNA and ctDNA parameters. [file MOL2-17-1844-s002.tif]

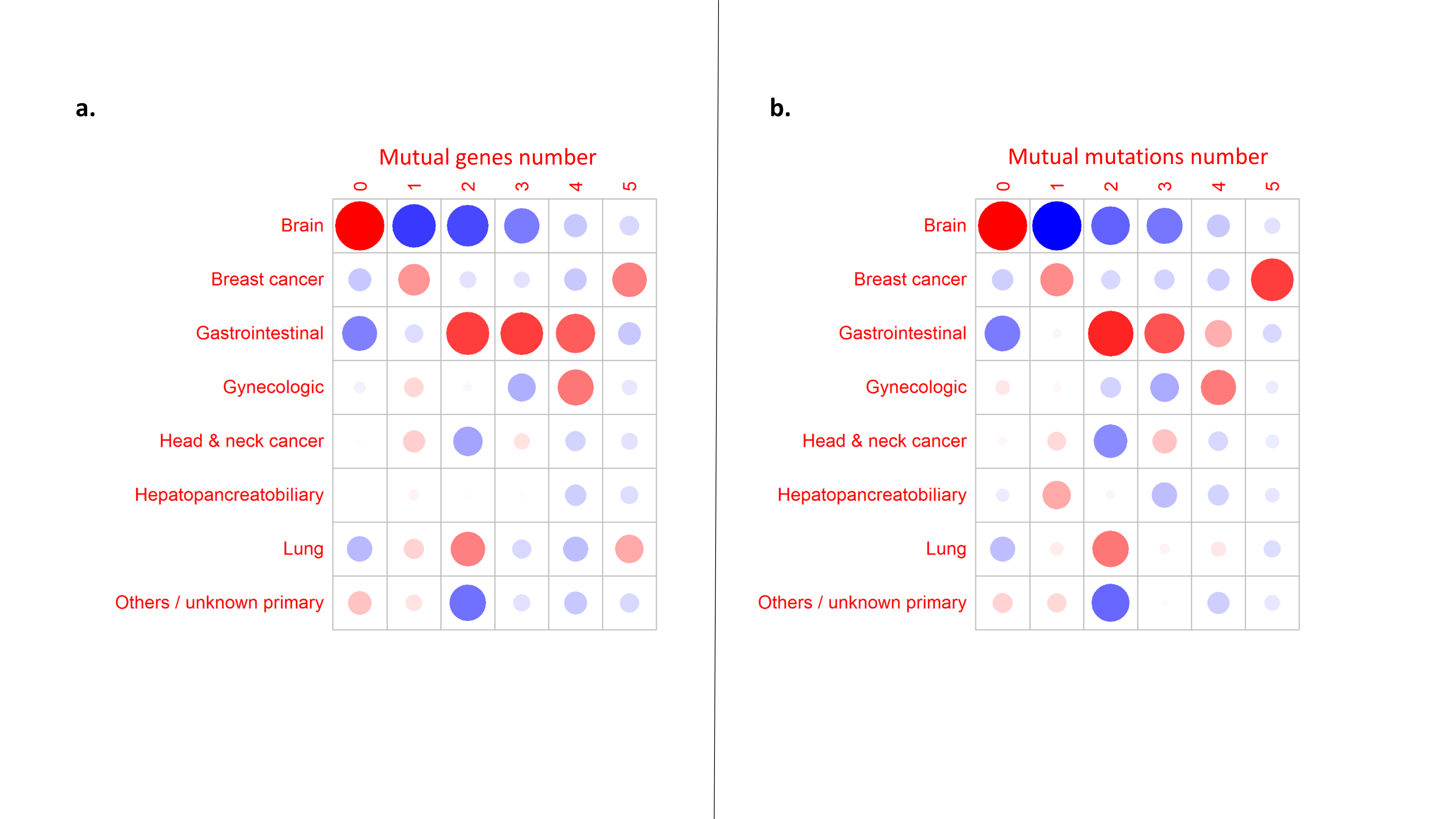

Supplement: Supplementary file 3 — Fig. S3. Balloon plots representing the residuals of the Chi‐square tests of: a. mutual gene concordance number tumor type; b. mutual mutation concordance number tumor type. [file MOL2-17-1844-s006.tif]

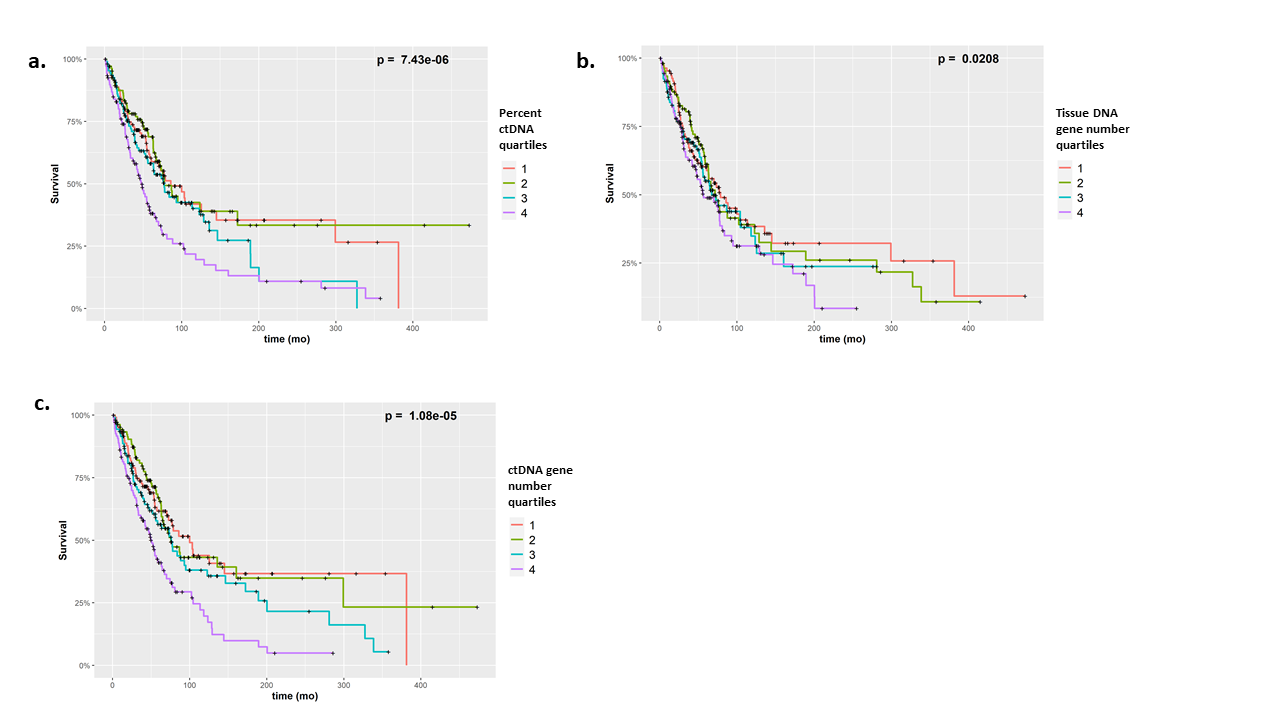

Supplement: Supplementary file 4 — Fig. S4. Univariate survival analysis of (a) quartiles of percent ctDNA, (b) quartiles of tissue DNA gene number, (c) quartiles of ctDNA gene number. [file MOL2-17-1844-s008.tif]

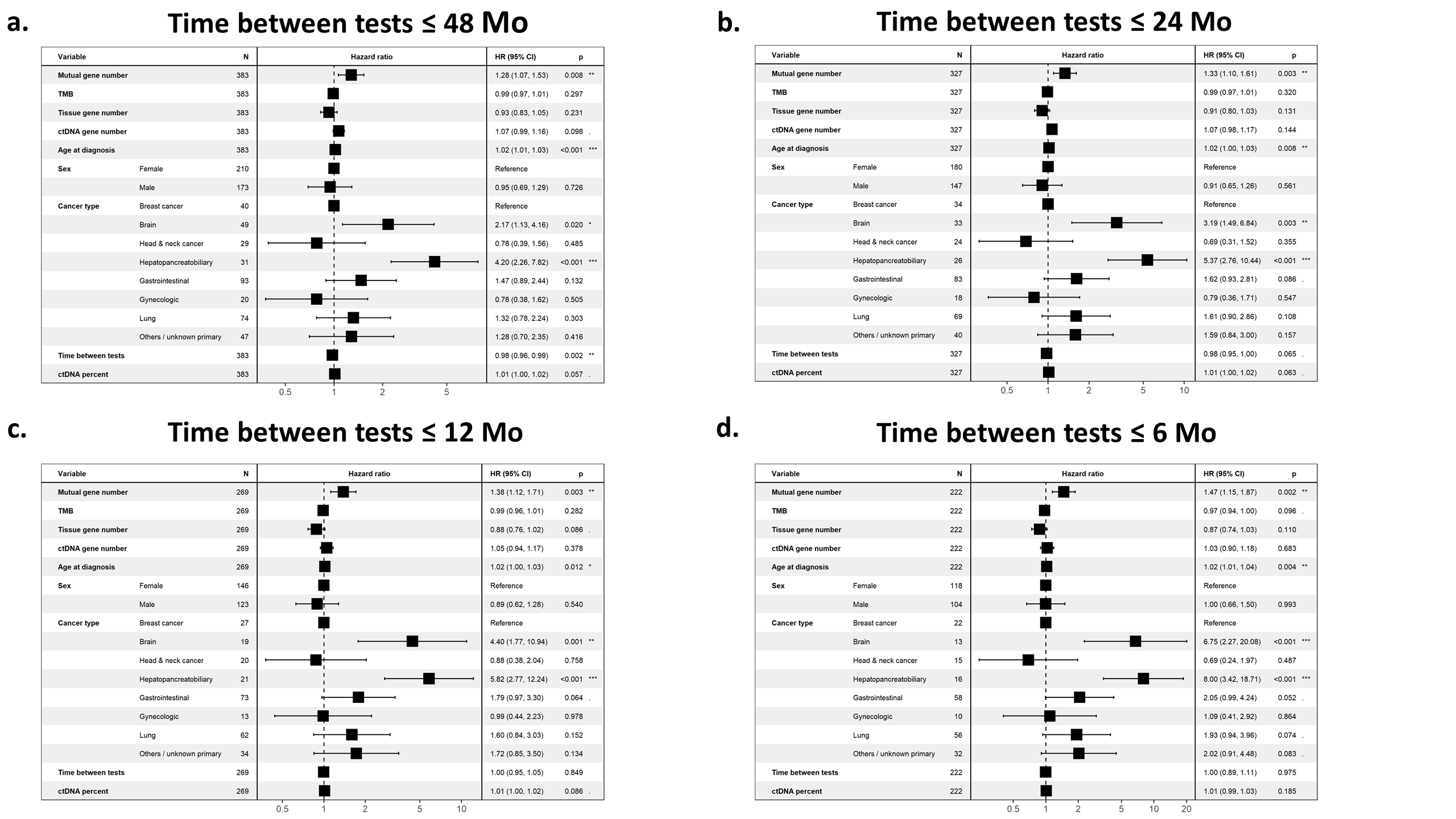

Supplement: Supplementary file 5 — Fig. S5. Multivariable analysis for the survival test in relation to the number of concordant genes (gene‐level concordance), similar to Fig. 2B including only samples for which the time gap between tissue DNA and ctDNA (a) ≤ 48 months, (b) ≤ 24 months, (c) ≤ 12 months, (d) ≤ 6 months. [file MOL2-17-1844-s001.tif]

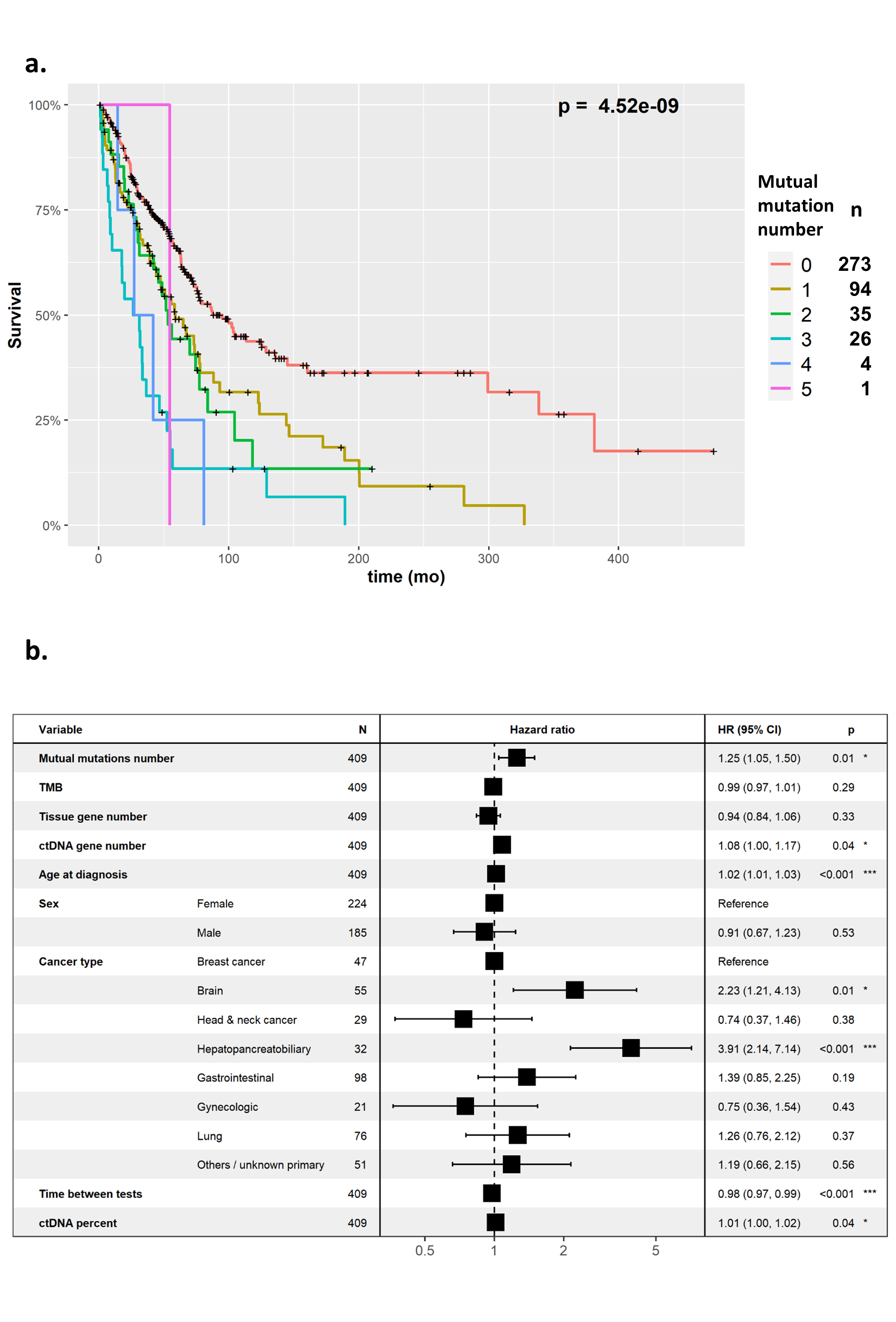

Supplement: Supplementary file 6 — Fig. S6. Survival analysis in relation to the number of concordant mutations (mutation‐level concordance). [file MOL2-17-1844-s004.tif]
